# Analysis of the Carry-Over of Ochratoxin A from Feed to Milk, Blood, Urine, and Different Tissues of Dairy Cows Based on the Establishment of a Reliable LC-MS/MS Method

**DOI:** 10.3390/molecules24152823

**Published:** 2019-08-02

**Authors:** Zhiqi Zhang, Zhichen Fan, Dongxia Nie, Zhihui Zhao, Zheng Han

**Affiliations:** Institute for Agro-Food Standards and Testing Technology, Shanghai Key Laboratory of Protected Horticultural Technology, Shanghai Academy of Agricultural Sciences, Shanghai 201403, China

**Keywords:** LC-MS/MS, dairy cows, ochratoxin A, ochratoxin α, carry-over

## Abstract

A rapid and reliable liquid chromatography-tandem mass spectrometry (LC-MS/MS) method was developed for simultaneous determination of ochratoxin A (OTA) and its metabolite ochratoxin α (OTα), for the first time, in dairy cow plasma, milk, urine, heart, liver, spleen, lung, and kidney. The established method was extensively validated by determining the linearity (R^2^ ≥ 0.990), sensitivity (lower limit of quantification, 0.1–0.2 ng mL^−1^), recovery (75.3–114.1%), precision (RSD ≤ 13.6%), and stability (≥83.0%). Based on the methodological advances, the carry-over of OTA was subsequently studied after oral administration of 30 μg/kg body weight OTA to dairy cows. As revealed, OTA and OTα were detected in urine, with maximal concentrations of 1.8 ng mL^−1^ and 324.6 ng mL^−1^, respectively, but not in milk, plasma, or different tissues, verifying the protection effects of rumen flora against OTA exposure for dairy cows. Moreover, 100 fresh milk samples randomly collected from different supermarkets in Shanghai were also analyzed, and no positive samples were found, further proving the correctness of the in vivo biotransformation results. Thus, from the currently available data, regarding OTA contamination issues on dairy cows, no significant health risks were related to OTA exposure due to the consumption of these products.

## 1. Introduction

Ochratoxin A (OTA), a secondary metabolite produced by the fungal genera *Aspergillus* and *Penicillium*, occurs in a variety of cereals and cereal products [1,2,3]. OTA can also be found in animal-derived-products (edible tissues, milk and eggs) due to the carry-over effects through the consumption of OTA-contaminated feeds, posing potential health risks to humans [4,5]. Being nephrotoxic, hepatotoxic, carcinogenic, teratogenic, and immunotoxic, OTA is classified as a possible human carcinogen (group 2B) by the International Agency for Research on Cancer (IARC) [1]. Taking into account all data currently available, the Panel on Contaminants in the Food Chain (CONTAM) of the European Food Safety Authority (EFSA) derived a Tolerable Weekly Intake (TWI) of 120 ng kg^−1^ body weight (b.w.) for OTA [6]. A Provisional Tolerable Weekly Intake (PTWI) of 100 ng kg^−1^ b.w. was established by a Joint FAO/WHO Expert Committee on Food Additives (JECFA) [6].

Due to its widespread occurrence and high toxicity, the in vivo kinetics and biotransformation mechanisms of OTA have attracted increasingly more attention. In general, kidney, liver, heart, lung, spleen, blood, urine, and milk (as the targeted biological matrix for this contaminant) are frequently investigated to reveal the in vivo kinetics and metabolism in different animals [4,7,8,9,10]. Up to now, the in vivo investigations of OTA have mainly focused on monogastric animals as a reference for humans [4,7,9,11,12]. Pigs were particularly sensitive to OTA due to its long plasma elimination half-life, and the highest accumulation was observed in kidneys, followed by liver, muscles, and fatty tissues [4]. In rats, OTA was slowly eliminated in plasma as well, while kidneys, heart, and liver were the targeted organs [9,12]. The primary metabolic pathway for OTA in different animals is the hydrolysis of the free form into a much less toxic metabolite ochratoxin α (OTα) by bacterial microflora. OTα, a dihydroisocoumarin derivative formed by the cleavage of the peptide bond of OTA, is easily eliminated from the body, posing an efficient approach for toxicity reduction [7]. Compared to monogastrics, dairy cows (the typical ruminants) are less susceptible due to an extensive pre-absorptive metabolism in rumen, where the rumen protozoa and bacteria could directly convert OTA into OTα [13,14,15]. Only a small amount of OTA was reported to be detected in blood after oral administration to dairy cows [8]. In contrast, in some other investigations, OTA could also be detected in milk, urine, and kidney [10,16,17]. So far, little information is available and remains controversial in this respect. It is, therefore, an important issue to determine the carry-over of OTA from feed to dairy cows.

To illuminate the biotransformation and distribution of OTA in dairy cows, the prerequisite is to establish an accurate and sensitive analytical method, which could be utilized for the determination of OTA and its major metabolite (OTα) in different biomatrices. Thin layer chromatography (TLC) was first employed to identify OTA, but its low sensitivity and unsatisfying accuracy limits its application [18]. Similarly, as a widely used approach for rapid OTA detection, enzyme-linked immunosorbent assay (ELISA) methods suffer from pseudo-positive and inaccurate results [19]. The most widely applied analytical tools for the accurate quantification of OTA in different sample matrices are high-performance liquid chromatography (HPLC) combined with different instruments, such as a fluorescence detector (FLD), mass spectrometry (MS), tandem mass spectrometry (MS/MS), nuclear magnetic resonance and mass spectrometry (NMR-MS), and high-resolution mass spectrometry (HRMS) [1,20,21]. Due to the simple sample preparation and high sensitivity and selectivity, liquid chromatography-tandem mass spectrometry (LC-MS/MS) appears to be the most promising tool and has been used for the in vivo detection of OTA in rats, chickens, and pigs [4,9,21]. However, to the best of our knowledge, there are still no uniform methods for simultaneous determination of OTA and OTα in plasma, milk, urine, and different tissues.

The major purpose of the present study was (1) to develop a rapid, accurate, sensitive, and selective LC-MS/MS method, which could be used for the detection of OTA and OTα in plasma, milk, urine, heart, liver, spleen, lung, and kidney and (2) to thoroughly investigate the carry-over of OTA from feed to biological fluids and tissues of dairy cows, so as to reveal the potential health risks to humans and animals due to the consumption of OTA-contaminated food and feed products.

## 2. Results

### 2.1. Optimization of Extraction Solvent

The peak area ratios of OTA and OTα (50 ng mL^−1^) spiked before extraction to those spiked after extraction represented the extraction recovery values. Three different solvents (methanol, acetonitrile and acetone) with different extraction volumes (0.6, 1.0, 1.4, 1.8 mL) were compared in the urine samples. As shown in Table S2, the extraction recoveries were in a range from 71.5% to 92.4%. The highest extraction efficiency, with recovery values of 89.8% for OTA and 92.4% for OTα, was achieved when 1.4 mL of acetone was selected.

### 2.2. LC-MS/MS Method Validation

**Figure 1:**
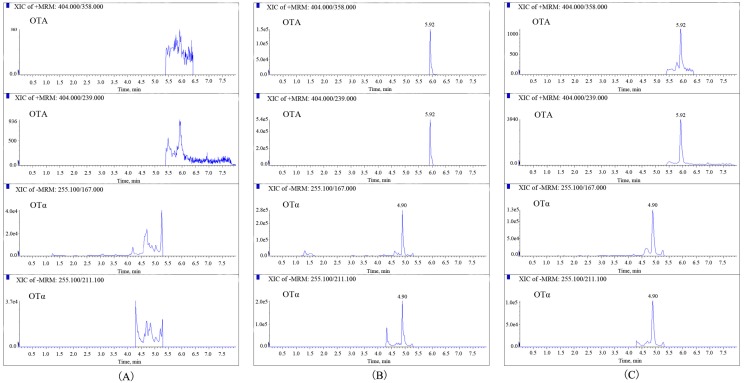
Multiple reaction monitoring (MRM) chromatograms of ochratoxin A (OTA) and ochratoxin α (OTα) in a blank urine sample (**A**), in spiked urine sample with the concentration of 20 ng mL^−1^ (**B**), and in urine samples collected at 12 h after oral administration of OTA in dairy cows (**C**).

The selectivity of the established method was first investigated. As shown in Figure 1, no interferences appeared at the retention time of OTA and OTα in blank urine samples. The same results were obtained for milk, plasma, and different tissue homogenates, demonstrating the optimal selectivity achieved for the detection of OTA and OTα in different biomatrices.

The calibration curves of OTA and OTα constructed in a neat solvent and various biomatrices are shown in Table 1. Good linear relationships with the coefficients of determination (R^2^) ≥0.990 were obtained. The lower limit of detection (LLOD) and lower limit of quantification (LLOQ) for OTA/OTα were in the range of 0.03–0.1 ng mL^−1^ and 0.1–0.2 ng mL^−1^, respectively (Table 1). High matrix effects were observed for the two analytes in different biomatrices with the signal suppression/enhancement (SSE) values in the range of 36–162%, and, as a consequence, the matrix-matched calibration curves were used for accurate quantification. The recoveries were in the range of 75.3–114.1% (Table 2), while the RSDs of OTA and OTα were in the ranges of 1.9–12.8% for intra-day precision and 6.1–13.6% for inter-day precision (Table 2). After careful investigation of the short-term stability (at RT for 8 h), long-term stability (at −20 °C for 20 days) and freeze-thaw stability (three circles), OTA and OTα were proven to be stable in all biomatrices under different conditions (≥83.0%), ensuring that no stability-related problems existed during the sample analysis (Table 3).

### 2.3. Method Application

The validated LC-MS/MS method was applied for the analysis of OTA and OTα in urine, milk, plasma, and various tissues (heart, liver, spleen, lung, and kidney). Further, a total of 100 milk samples randomly collected from different supermarkets were analyzed. The results showed that OTA and OTα were not found in all milk samples, nor in plasma or tissues. With regard to urine, after oral administration, most of the samples were positive for OTA and OTα. OTα was the major excretory product, with concentrations in the range of 84.2–324.6 ng mL^−1^, while trace amounts (0.6–1.8 ng mL^−1^) of OTA were also found. The highest concentrations of OTA and OTα were achieved, with Tmax being 720 min (Table 4). Multiple reaction monitoring (MRM) chromatograms of OTA and OTα in neat solvents and in urine samples collected at 720 min after oral administration of OTA in dairy cows are shown in Figure 1.

## 3. Discussion

In the current work, a rapid and reliable LC-MS/MS method was established to reveal, for the first time, the carry-over of OTA from feed to milk, blood, urine, and different tissues of dairy cows. The method has been thoroughly validated by determining its selectivity, linearity, sensitivity, recovery, precision, and stability. As shown in Table 5, the sensitivities of the current established method were comparable or even higher than those of the previous studies on cows [8,10,16,17]. All parameters clearly indicated that the established method was selective, accurate, and reproducible, and thus could be applied to simultaneous analyses of OTA and OTα in various biomatrices.

Since cow milk is the main milk type used for human consumption, accounting for 83% of world milk production [22], most attention has been paid to the transformation of OTA from feed to cow milk [8,23,24]. Herein, OTA and OTα were not detected in any milk sample, which was similar to some previous studies showing that barely detectable amounts of OTA were recovered into cow milk [8,10,25], and also rarely into dairy sheep milk [26]. In contrast, considerable amounts of OTA and OTα were found in the milk of one cow, but only following the administration of a large dose (13.3 mg/kg b.w.) of OTA [16]. The low carry-over of OTA might be due to rumen microbiota, which could effectively degrade OTA to the less toxic OTα, which would then be excreted to urine [13,14,15]. Moreover, OTA and OTα were not detected in 100 fresh milk samples randomly collected from different supermarkets in Shanghai, further proving the reliability of the established LC-MS/MS and the correctness of the in vivo biotransformation results. Therefore, currently, no health risks related to OTA contamination could be posed to humans due to the consumption of milk in Shanghai, China. However, the rumen microflora with the cleavage capacity of OTA could be significantly influenced by feed compositions and the animals’ physical conditions [27,28]. Thus, in some studies, OTA could also be found in cow’s milk samples and dairy products [23,29]. Consequently, continuously monitoring OTA in milk is of great importance to ensure its safety.

The metabolic parameters of OTA could strongly influence its effects on animal health. The kinetics and biotransformation of OTA in humans or monogastric animals indicate that OTA can be detected in plasma and/or different tissues, especially in kidneys [4,9]. On the contrary, in the present study, no measurable level of the OTA and OTα was found in plasma, heart, liver, spleen, lung, or kidney in dairy cows, indicating that before any essential uptake, OTA has been subjected to microbial degradation in ruminants [13,14,15,28]. These observations were consistent with the reported literature [8,10]. In young (pre-ruminant) calves, a plot of the serum OTA concentration–time data is likely related to the incomplete development of rumen function [17]. The development of a functional rumen greatly influenced the tolerance of calves/cows to orally administered OTA. All the above results proved that rumen functionality represented the main barrier to the passage of OTA into the milk, bloodstream, and different edible tissues, thus preventing OTA in cow products from being an important risk factor for humans.

Until now, the transformation of OTA from feeds to cow urine has not been well defined. As reported, after administration of OTA to Jersey milking cows, the urine was free of OTA and the metabolite OTα [10]. However, contrary to these negative results, according to our analysis, high amounts of OTα and trace amounts of OTA were detected in urine. These differences may be attributed to the LC-MS/MS method established in the current study, which clearly has a higher LLOQ (0.1 ng mL^−1^) compared to the previous study (detection limit of 5 ng mL^−1^). Our positive results were in good agreement with some other previous studies, in which OTA was proven to be mainly metabolized into OTα and excreted in urine after being ingested by dairy cows [16,17].

## 4. Materials and Methods

### 4.1. Reagents and Chemicals

The standards of OTA and OTα were acquired from Sigma–Aldrich (St. Louis, MO, USA). The structures of these two mycotoxins are shown in Table S1. Acetonitrile and methanol were purchased from Merck (Darmstadt, Germany). Water used throughout the analyses was Milli-Q quality water (Millipore, Billerica, MA, USA). All reagents and chemicals were analytical or HPLC grade.

### 4.2. Apparatus

LC-MS/MS was utilized for the analysis. OTA and OTα were separated via a Waters ACQUITY UPLC system (Waters, Milford, MA, USA) at 40 °C using a Poroshell EC-C18 column (100 mm × 3.0 mm, i.d. 2.7 μm) (Agilent, Santa Clara, CA, USA). A linear gradient elution using (A) methanol and (B) water (containing 5 mmol L^−1^ ammonium acetate) as the mobile phase was as follows: initial 0–0.5 min 10% A, 0.5–6 min 10% A, 6–6.5 min 90% A, 6.5–6.7 min 10% A, 6.7–8 min 10% A, and held on for another 3 min for equilibration. The mobile phase flow rate was 0.4 mL min^−1^ with an injection volume of 3 μL.

MS/MS analysis was performed on a Triple-Quad™ 5500 mass spectrometer (AB Sciex, Foster City, CA, USA) with the electrospray ionization source operating in both positive (ESI^+^) and negative (ESI^−^) modes. The parameters were set as follows: desolvation temperature of 500 °C; source temperature of 150 °C; cone gas flow of 7 L h^−1^; desolvation gas flow of 1000 L h^−1^. The MRM mode was used for the quantification of OTA and OTα. The primary and secondary transitions (*m*/*z*) were 404.2 → 239 (collision energy of 30 eV), 404.2 → 358 (collision energy of 26 eV) for OTA and 255.1 → 167 (collision energy of −30 eV), 255.1 → 211.1 (collision energy of −30 eV) for OTα, respectively. Data were processed by the MultiQuant algorithm from MultiQuant 3.0.2 (Analyst; AB SCIEX, Foster City, CA, USA).

### 4.3. Diets and Animals

This study was approved by the Animal Ethics Committee in Shanghai Academy of Agricultural Science(Shanghai, China) (SYXK (Hu) 2015-0007) and Zhuozhou Jierong Bio-Technology Co., Ltd. (Zhuozhou City, Hebei, China)(SYXK (Ji) 2018-003) in accordance with Chinese guidelines for animal welfare.

Five healthy Holstein cows in lactation with similar initial body conditions (500 ± 8.6 kg of b.w.) from Zhangxueping Dairy Farm (Nanjing, China) were randomly divided into 2 groups (one cow for the control group and four cows for the experimental group). The cows were fed 20 kg total mixed rations (TMR) feed (7:00; 17:00) and milk (7:30; 17:30) twice a day, and the milk yield was recorded. After an overnight fast, 7:00 in the morning was the specific exposure time for OTA to the dairy cows. At this time, the animals were orally given a single dose of the 250 g OTA-containing (30 μg kg^−1^ b.w.) TMR feed (experimental group) and OTA-free TMR feed (control group), respectively”.

### 4.4. Sample Collection

The samples of milk, blood, urine, and different tissues gathered from the control group were used to establish the analytical method, while the samples from experimental group were used for OTA and OTα measurements. The daily milk collected from the morning and evening was homogenized and stored at −20°C until analysis. Blood samples were individually collected from each cow via caudal vein into heparinized tubes at 10, 35, 45, 60, 120, 180, 240, 360, 540, 720, 1440, 2160, and 2880 min after OTA ingestion. After centrifugation (3500× *g*, 15 min, 4 °C), the supernatant was transferred into clean tubes and stored at −20 °C until analysis. After OTA administration, urine samples were collected over a period of 3 d, following which, OTA and OTα could no longer be detected in urine. After these experiments, the experimental group was fed with OTA-free TMR feed for one month, to guarantee OTA disappearance in the body, and then orally given OTA-containing TMR feed again. All cows were sacrificed 6 h after OTA oral administration and the heart, liver, spleen, lung, and kidney samples were collected. The samples were individually homogenized with normal saline (*m*/*v*, 1/3, 0 °C) and stored at −20 °C until analysis.

On the other hand, a total of 100 fresh milk samples were randomly collected from different supermarkets in Shanghai. All the samples were stored at −20 °C until analysis.

### 4.5. Sample Pretreatment

To the milk, plasma, urine, or tissue homogenates (200 μL), 800 μL of methanol was added for participation of the proteins. After vortex shaking for 30 s and centrifugation (12,000× *g*, 4 °C) for 5 min, the supernatant was transferred into 1.5 mL clean tubes and then dried by nitrogen gas at 40 °C. The residues were re-dissolved in 200 μL 5 mmol L^−1^ of ammonium acetate/acetonitrile (80/20, *v*/*v*), passed through the syringe filters (0.22 μm), and prepared for LC-MS/MS analysis.

### 4.6. LC-MS/MS Method Validation

The performance of the analytical method, including the linearity, specificity, sensitivity, accuracy, and repeatability, were validated for each matrix (i.e., milk, urine, plasma, and various tissues).

The selectivity of the analytical method was investigated by comparing MRM chromatograms of the blank samples, the blank samples spiked with OTA and OTα (20 ng mL^−1^), and the samples collected from OTA-treated cows. Calibrations curves (1/x weighted) were created for OTA and OTα by plotting the responses versus the concentrations (0.1, 0.2, 0.5, 1, 2, 5, 10, 20, 50, 100, and 200 ng mL^−1^) in plasma, milk, urine, heart, liver, spleen, lung, kidney, and neat solvent (5 mmol L^−1^ of ammonium acetate/acetonitrile (80/20, *v*/*v*)), respectively. The matrix effect was represented by SSE and calculated by comparing the slope of the matrix-matched calibration curves with the slope of calibration built in the neat solvent. The sensitivity was evaluated in terms of LLOD and LLOQ, which were designed as the levels of the compounds that yield a signal-to-noise (S/N) of 3 and 10 in each matrix, respectively.

Blank samples spiked with LLOQ, with low (1 ng mL^−1^), intermediate (50 ng mL^−1^), and high (200 ng mL^−1^) concentrations of OTA and OTα, were used to perform the recovery, intra-and inter-day precision investigations (*n* = 6). Recovery was calculated by comparing the determined concentrations with the spiked concentrations. The same spiked samples in one day and in five consecutive days were used to evaluate the intra-day and inter-day precisions. To investigate stability-related matters during the routine and extensive analysis of samples, the short-term, long-term, and freeze-thaw stabilities of OTA and OTα were assessed by comparing the concentrations of OTA and OTα in the spiked samples (*n* = 6) stored at room temperature (RT) for 8 h , at −20 °C for 20 days and subjected to three freeze-thaw cycles with that in the samples freshly prepared respectively.

## 5. Conclusions

The current study developed a rapid and reliable LC-MS/MS method for quantitative analysis of OTA and OTα in dairy cow milk, plasma, urine, heart, liver, spleen, lung, and kidney. This well-established method with good performances (selectivity, linearity, sensitivity, recoveries, precisions, and stability) was successfully applied to determine the carry-over of OTA after oral administration of the toxin to dairy cows. As revealed, due to the degradation by rumen microbiota, OTA and OTα were only found in urine samples, but not in milk, plasma, or different tissues, thereby precluding the significant health risks correlated with OTA exposure by the consumption of these products in Shanghai, China.

## Figures and Tables

**Table 1:** Linearity, lower limit of detection (LLOD), and lower limit of quantification (LLOQ), matrix effect (SSE) of ochratoxin A (OTA) and ochratoxin α (OTα).

Matrix	Mycotoxin	Linear Range (ng mL^−1^)	Slope	Intercept	R^2^	LLOD (ng mL^−1^)	LLOQ (ng mL^−1^)	SSE (%)
Solvent	OTA	0.1–200	12,601	275	0.999	/	/	/
	OTα	0.1–200	37,500	846	0.999	/	/	/
Milk	OTA	0.1–200	20,432	542	0.994	0.03	0.1	162
	OTα	0.1–200	21,438	1215	0.999	0.03	0.1	57
Urine	OTA	0.2–200	14,564	180	0.990	0.1	0.2	116
	OTα	0.2–200	13,428	197	0.990	0.1	0.2	36
Plasma	OTA	0.1–200	26,422	−2142	0.995	0.03	0.1	130
	OTα	0.1–200	27,476	2219	0.997	0.03	0.1	73
Heart	OTA	0.1–200	6378	810	0.997	0.03	0.1	51
	OTα	0.1–200	30,231	2037	0.994	0.03	0.1	81
Liver	OTA	0.1–200	5598	1109	0.999	0.03	0.1	44
	OTα	0.1–200	24,216	−501	0.997	0.03	0.1	65
Spleen	OTA	0.1–200	4990	636	0.998	0.03	0.1	40
	OTα	0.1–200	26,424	1424	0.993	0.03	0.1	70
Lung	OTA	0.1–200	4934	617	0.999	0.03	0.1	39
	OTα	0.1–200	22,983	1086	0.998	0.03	0.1	61
Kidney	OTA	0.1–200	5048	851	0.998	0.03	0.1	40
	OTα	0.1–200	26,374	700	0.995	0.03	0.1	70

**Table 2:** Recoveries, intra-and inter-day precisions of ochratoxin A (OTA), and ochratoxin α (OTα) in different matrices (*n* = 6).

Matrix	Mycotoxin	Spiked Level (ng mL^−1^)	Recovery (Mean ± SD, %)	Intra-Day Precision (RSD, %)	Inter-Day Precision (RSD, %)
Milk	OTA	LLOQ	78.6 ± 4.0	5.0	10.7
		1	95.4 ± 10.7	11.2	12.0
		50	86.3 ± 5.8	6.7	11.0
		200	80.1 ± 8.1	10.1	11.3
	OTα	LLOQ	106.7 ± 5.3	4.9	12.2
		1	88.9 ± 9.4	10.6	11.9
		50	94.6 ± 8.0	8.5	12.1
		200	100.3 ± 3.5	3.5	8.6
Urine	OTA	LLOQ	75.3 ± 4.1	5.5	12.6
		1	82.4 ± 5.2	6.3	11.3
		50	87.4 ± 6.1	6.9	10.8
		200	88.7 ± 6.0	6.8	9.9
	OTα	LLOQ	99.3 ± 6.4	6.5	10.5
		1	83.8 ± 8.1	9.6	12.2
		50	79.3 ± 5.8	7.3	9.0
		200	84.3 ± 6.8	8.1	11.7
Plasma	OTA	LLOQ	87.9 ± 9.7	11.0	13.4
		1	84.9 ± 7.3	8.6	11.3
		50	83.5 ± 8.8	10.6	10.6
		200	75.6 ± 4.5	5.9	10.1
	OTα	LLOQ	114.1 ± 2.2	1.9	10.3
		1	87.9 ± 8.6	9.8	12.9
		50	97.3 ± 4.4	4.5	6.1
		200	92.6 ± 11.8	12.8	13.6
Heart	OTA	LOQ	112.1 ± 4.9	4.4	8.6
		1	94.0 ± 7.5	8.0	11.9
		50	91.1 ± 9.4	10.4	11.8
		200	99.0 ± 3.6	3.7	8.6
	OTα	LLOQ	101.1 ± 5.9	5.8	12.4
		1	87.0 ± 9.7	11.2	12.0
		50	96.8 ± 9.0	9.3	11.7
		200	89.2 ± 9.1	10.2	10.3
Liver	OTA	LLOQ	101.1 ± 6.3	6.3	10.2
		1	91.0 ± 6.5	7.1	10.3
		50	102.1 ± 6.8	6.7	11.9
		200	91.9 ± 9.7	10.6	12.3
	OTα	LLOQ	81.6 ± 6.9	8.4	11.6
		1	94.7 ± 5.2	5.5	9.7
		50	102.8 ± 5.0	4.9	9.3
		200	95.4 ± 4.3	4.5	10.8
Spleen	OTA	LLOQ	109.8 ± 8.0	7.3	7.9
		1	97.8 ± 6.3	6.5	12.5
		50	91.1 ± 2.1	2.3	11.4
		200	96.7 ± 5.6	5.8	10.4
	OTα	LLOQ	105.6 ± 4.3	4.1	12.1
		1	99.4 ± 8.9	8.9	9.7
		50	93.0 ± 6.5	7.0	12.0
		200	90.1 ± 6.4	7.1	10.9
Lung	OTA	LLOQ	99.0 ± 4.9	5.0	8.0
		1	89.6 ± 9.1	10.2	11.6
		50	89.0 ± 8.0	8.9	9.8
		200	85.3 ± 5.0	5.8	10.1
	OTα	LLOQ	107.5 ± 6.4	5.9	10.5
		1	97.7 ± 3.8	3.9	9.4
		50	94.0 ± 5.7	6.0	11.4
		200	98.0 ± 5.8	5.9	11.6
Kidney	OTA	LLOQ	105.2 ± 8.0	7.6	12.6
		1	91.0 ± 5.4	6.0	11.2
		50	99.5 ± 2.5	2.6	10.9
		200	88.1 ± 9.1	10.4	12.8
	OTα	LLOQ	108.9 ± 6.3	5.8	10.8
		1	90.9 ± 10.7	11.8	11.6
		50	94.1 ± 7.8	8.3	12.9
		200	87.4 ± 8.7	9.9	10.2

“LLOQ”: lower limit of detection.

**Table 3:** Stability of ochratoxin A (OTA) and ochratoxin α (OTα) in different biomatrices (*n* = 6).

Matrix	Mycotoxin	Spiked Level (ng mL^−1^)	Recovery (Mean ± SD %)		
			Short-Term Stability ^a^	Freeze-Thaw Stability ^b^	Long-Term Stability ^c^
Urine	OTA	1	98.3 ± 8.1	97.6 ± 6.1	98.7 ± 6.7
		50	101.4 ± 2.4	102.2 ± 4.9	99.8 ± 3.6
	OTα	1	89.6 ± 2.9	88.3 ± 7.9	87.9 ± 8.1
		50	98.5 ± 7.0	93.6 ± 4.6	92.3 ± 4.8
Plasma	OTA	1	86.0 ± 7.8	113.6 ± 2.8	93.4 ± 7.4
		50	90.7 ± 11.8	93.2 ± 7.3	86.7 ± 6.7
	OTα	1	96.2 ± 11.7	94.7 ± 8.5	96.9 ± 7.8
		50	88.6 ± 5.5	96.7 ± 9.3	94.4 ± 4.6
Heart	OTA	1	90.5 ± 8.6	95.3 ± 8.6	91.1 ± 5.1
		50	85.8 ± 7.1	91.3 ± 9.1	99.0 ± 11.9
	OTα	1	99.0 ± 8.4	86.3 ± 10.4	95.5 ± 12.4
		50	91.3 ± 11.6	90.8 ± 7.2	97.1 ± 8.6
Liver	OTA	1	91.0 ± 9.4	98.0 ± 10.1	93.5 ± 9.5
		50	90.5 ± 8.2	94.2 ± 10.0	92.1 ± 10.3
	OTα	1	92.4 ± 7.2	92.6 ± 10.4	98.3 ± 9.4
		50	95.3 ± 8.5	93.3 ± 6.8	98.9 ± 9.6
Spleen	OTA	1	87.7 ± 12.3	84.3 ± 6.1	90.0 ± 9.9
		50	96.8 ± 10.1	88.8 ± 5.1	97.9 ± 5.5
	OTα	1	92.0 ± 12.6	96.2 ± 12.1	83.0 ± 5.1
		50	94.4 ± 6.1	98.5 ± 9.8	92.5 ± 9.4
Lung	OTA	1	86.1 ± 8.1	92.9 ± 9.7	93.3 ± 9.8
		50	90.9 ± 7.7	97.0 ± 11.4	85.1 ± 3.9
	OTα	1	89.6 ± 8.2	94.4 ± 10.9	98.1 ± 8.3
		50	93.7 ± 9.8	96.9 ± 10.2	85.3 ± 10.8
Kidney	OTA	1	87.8 ± 9.4	87.3 ± 9.1	93.8 ± 6.0
		50	100.4 ± 7.4	86.6 ± 8.7	90.6 ± 10.0
	OTαα	1	89.3 ± 9.7	97.2 ± 8.1	92.7 ± 9.7
		50	98.2 ± 8.3	91.8 ± 8.9	99.0 ± 6.0

The short-term, the freeze-thaw and long-term stabilities of OTA and OTα were assessed by comparing the concentrations of OTA and OTα in the spiked samples stored at different conditions with those in the freshly prepared samples: “a”: stored at room temperature (RT) for 8 h; “b”: subjected to three freeze-thaw cycles; “c”: stored at −20 °C for 20 days.

**Table 4:** The concentrations of ochratoxin A (OTA) and ochratoxin α (OTα) in urine samples collected at different times after oral administration of OTA in dairy cows (ng mL^−1^) (*n* = 4).

Time (min)	OTA	OTα
120	-	-
360	1.6 ± 0.12	203.8 ± 16.3
720	1.8 ± 0.09	324.6 ± 23.2
1440	1.0 ± 0.06	232.6 ± 13.9
2160	0.6 ± 0.07	141.2 ± 11.3
2880	-	84.2 ± 7.6
4320	-	-

“-”: not detected.

**Table 5:** Comparison of previous studies for the determination of ochratoxin A (OTA) and ochratoxin α (OTα) in different biosamples from cows.

Animals	Dosage	Mycotoxin/Administration	Matrices	Analytical Method	LLOD (ng mL^−1^)		LLOQ (ng mL^−1^)		Results	Reference
					OTA	OTα	OTA	OTα		
Jersey milking cows	317–1125 μg/kg diets	Naturally contaminated barley containing OTA/Feeding	Milk, serum, urine, and tissues ^a^	TLC	5	5	/	/	OTA(5 μg/kg) was detected in kidney	[10]
Holstein cows	0.2–13.3 mg/kg b.w.	Pure OTA/By stomach	Milk and urine	TLC	/	/	/	/	OTA and OTα were detected in milk and urine ^d^	[16]
Young Holstein-Friesian male calves	0.25–2 mg/kg b.w.	Pure OTA/Feeding	Serum, urine, and feces	HPLC	50	50	/	/	OTA and OTα were detected in serum, urine and feces ^e^	[17]
Holstein cows	5, 50 or 100 μg/kg diets	Pure OTA/Feeding	Plasma, milk, and tissues ^b^	HPLC	/	/	0.1	/	OTA (0.1 μg/kg) was detected in the plasma	[8]
Holstein cows	30 μg/kg b.w.	Pure OTA/ Feeding	Plasma, milk, urine, and tissues ^c^	HPLC-MS/MS	0.03–0.1	0.03–0.1	0.1–0.2	0.1–0.2	OTA and OTα were detected in urine	This study

“a”: Muscle, liver, and kidney; “b”: Liver, muscles, and fat; “c”: Heart, liver, spleen, lung, and kidney; “d”: OTA occurred in cow’s milk and urine but only when massive doses were ingested. All cows had traces of OTα in milk and urine; “e”: Approximately 90% of the OTA was excreted as OTα, OTA was low in the urine or feces. OTA was absorbed rapidly into serum; b.w.: body weight. “TLC”: Thin-layer chromatography; “HPLC”: High-performance liquid chromatography; “HPLC-MS/MS”: High-performance liquid chromatography-tandem mass spectrometry.

## Supplementary Materials

The following are available online. Tables S1 and S2.

## Abbreviations

The following abbreviations are used in this manuscript:

b.w.	Body weight
CONTAM	Contaminants in the Food Chain
ELISA	Enzyme-linked immunosorbent assay
EFSA	European Food Safety Authority
FLD	Fluorescence detector
HPLC	High-performance liquid chromatography
HPLC-MS/MS	High-performance liquid chromatography-tandem mass spectrometry
HRMS	High-resolution mass spectrometry
IARC	International Agency for Research on Cancer
JECFA	Joint FAO/WHO Expert Committee on Food Additives
LC-MS/MS	Liquid chromatography-tandem mass spectrometry
LLOD	Lower limit of detection
LLOQ	Lower limit of quantification
MRM	Multiple reaction monitoring
MS	Mass spectrometry
NMR-MS	Nuclear magnetic resonance and mass spectrometry
OTA	Ochratoxin A
OTα	Ochratoxin α
PTWI	Provisional tolerable weekly intake
RT	Room temperature
SSE	Signal suppression/enhancement
TLC	Thin-layer chromatography
TMR	Total mixed rations
MS/MS	Tandem mass spectrometry
TWI	Tolerable Weekly Intake
