# Supplementary material for: Analysis of the Carry-Over of Ochratoxin A from Feed to Milk, Blood, Urine, and Different Tissues of Dairy Cows Based on the Establishment of a Reliable LC-MS/MS Method
Source: Molecules. 2019 Aug 2;24(15):2823. doi: 10.3390/molecules24152823 (PMC6695942; doi:10.3390/molecules24152823)
Supplement: Supplementary file 1 [file molecules-24-02823-s001.pdf]

Table S1. The molecular weights, molecular formulas, and chemical structures of ochratoxin A (OTA) and ochratoxin α (OTα).

Mycotoxin	Molecular Weight	Molecular Formula	Chemical Structure
OTA [303-47-9]	403.81	$C_{20}H_{18}O_6ClN$	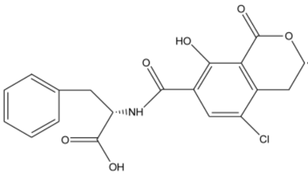
OTα [19165-63-0]	256.64	$C_{11}H_9O_5Cl$	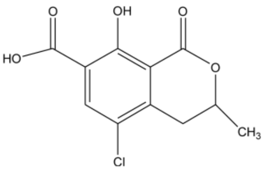

Table S2 Extraction recoveries of ochratoxin A (OTA) and ochratoxin α (OTα) from spiked urine samples (50 ng mL^{-1}) using different extraction solvents (%) (n = 6).

Extraction solvent (mL)	OTA	OTα
Methanol (0.6)	74.6±3.0	71.5±2.8
Methanol (1.0)	79.6±3.9	77.4±6.2
Methanol (1.4)	82.4±4.1	79.8±5.6
Methanol (1.8)	80.1±4.8	73.2±5.6
Acetonitrile (0.6)	79.3±3.2	82.4±6.9
Acetonitrile (1.0)	82.4±4.1	83.6±7.5
Acetonitrile (1.4)	87.8±5.3	85.7±6.9
Acetonitrile (1.8)	81.4±2.4	84.2±7.6
Acetone (0.6)	80.3±3.2	81.3±9.3
Acetone (1.0)	83.6±4.8	87.5±7.9
Acetone (1.4)	89.8±2.7	92.4±8.5
Acetone (1.8)	81.3±3.2	89.6±7.2
